# A new species of *Diploderma* (Reptilia, Squamata, Agamidae) from eastern Xizang, China

**DOI:** 10.3897/zookeys.1296.198499

**Published:** 2026-11-06

**Authors:** Shuo Liu, JiShan Wang, Mian Hou, Wei Zeng, Hongyuan Zhang, Dingqi Rao

**Affiliations:** 1 Kunming Natural History Museum of Zoology, Kunming Institute of Zoology, Chinese Academy of Sciences, Kunming, Yunnan 650223, China Kunming Natural History Museum of Zoology, Kunming Institute of Zoology, Chinese Academy of Sciences Kunming China; 2 Key Laboratory of National Forestry and Grassland Administration on Biodiversity Conservation on the Qinghai-Xizang Plateau, Kunming Institute of Zoology, CAS, Kunming, Yunnan 650201, China Key Laboratory of National Forestry and Grassland Administration on Biodiversity Conservation on the Qinghai-Xizang Plateau, Kunming Institute of Zoology Kunming China; 3 Southwest Survey and Planning Institute of National Forestry and Grassland Administration, Kunming, Yunnan 650031, China Southwest Survey and Planning Institute of National Forestry and Grassland Administration Kunming China; 4 College of Continuing (Online) Education, Sichuan Normal University, Chengdu, Sichuan 610066, China College of Continuing (Online) Education, Sichuan Normal University Chengdu China; 5 Kunming Institute of Zoology, Chinese Academy of Sciences, Kunming, Yunnan 650201, China Kunming Institute of Zoology, Chinese Academy of Sciences Kunming China

**Keywords:** Morphology, mountain lizard, ND2, Nujiang River system, phylogeny, taxonomy

## Abstract

A new species of *Diploderma* is described from the valley of a tributary of the Nujiang River in eastern Xizang, China, based on morphological and genetic data. The new species is morphologically most similar and phylogenetically closely related to *D. laeviventre* and *D. yangi*, but it can be distinguished from the latter two species by having a differently colored gular spot in both sexes and by several morphometric differences. This study brings the total number of species within the genus *Diploderma* to 51, of which four inhabit the Nujiang River system.

## Introduction

The genus *Diploderma* Hallowell, 1861 is a group of lizards with the majority of its species endemic to China (Uetz et al. 2026). Some species of this genus inhabit areas outside the Hengduan Mountain region and have wide distributions, while most species of this genus inhabit the river valleys of the Hengduan Mountain region and their distribution ranges are very limited (Wang et al. 2018, 2019b, 2019c, 2020, 2021; Che et al. 2021; Cai et al. 2022, 2024; Liu et al. 2022, 2023, 2024, 2025; Cai 2023). Among the numerous river valleys in the Hengduan Mountain region, three species of *Diploderma* are currently known to inhabit the Nujiang River valley, namely *D. laeviventre* (Wang, Jiang, Siler & Che, 2016), *D. slowinskii* (Rao, Vindum, Ma, Fu & Wilkinson, 2017), and *D. yangi* Wang, Zhang & Li, 2022. Currently, it is known that *D. laeviventre* is only found in the section of the Nujiang River valley near Basu County, Changdu City, Xizang Autonomous Region, *D. yangi* is only found in the downstream section of the Nujiang River valley near Chawalong Township, Chayu County, Linzhi City, Xizang Autonomous Region, and *D. slowinskii* is found further downstream of the Nujiang River valley in Nujiang Prefecture of Yunnan Province (Wang et al. 2022; Uetz et al. 2026).

During our 2020 field survey in Chayu County, Linzhi City, Xizang Autonomous Region, specimens of *Diploderma* were collected from the valley of a tributary of the Nujiang River in Wabu Village of Chawalong Township. Morphologically, these specimens are similar to *D. laeviventre* and *D. yangi* but also have some differences from them. Based on subsequent comprehensive analyses combining molecular systematics and morphology, we confirmed that these specimens belonged to an unnamed species. Here, we describe them as a new species of *Diploderma*.

## Material and methods

The specimens were collected between 1:00 p.m. and 6:00 p.m. and photographed alive prior to euthanasia. The collected specimens were preserved in 75% ethanol for permanent storage and deposited in the Kunming Natural History Museum of Zoology, Kunming Institute of Zoology, Chinese Academy of Sciences (KIZ).

Measurements were taken to the nearest 0.1 mm with digital vernier calipers following Wang et al. (2022); paired pholidosis characteristics were recorded in left/right:

**CR** canthus rostralis scale count, number of elongated, overlapping scales from posterosuperior nasal to and including last supraciliary scale along canthal ridge

**F4S** finger IV subdigital lamellae count, number of subdigital lamellae scale from base between fingers III and IV to tip of finger IV, excluding claw

**FLL** forelimb length, from axillary region to tip of finger IV, excluding claw

**HD** head depth, from top of head to base of mandible at temporal region of head

**HL** head length, from tip of snout to jaw joint

**HLL** hindlimb length, from groin region to tip of toe IV, excluding claw

**HW** head width, between widest points of head posterior to eyes

**IL** infralabial scale count, number of enlarged, modified labial scales from mental to corner of mouth

**MD** middorsal crest scale count, number of modified crest scales longitudinally from first nuchal crest to scale above cloaca

**NSL** nasal–supralabial scale rows, number of horizontal rows of small scales between first supralabial and nasal

**SEL** snout–orbit length, from tip of snout to anterior edge of orbital bone

**SL** supralabial scale count, number of enlarged, modified labial scales from rostral to corner of mouth

**SOR** number of horizontal rows of scales between supralabials and orbit circle

**SVL** snout–vent length, from snout tip to anterior edge of cloaca

**T4L** Toe IV length, from tip of toe IV to base between toes III and IV, excluding claw

**T4S** toe IV subdigital lamellae count, number of subdigital lamellae from base between toes III and IV to tip of toe IV, excluding claw

**TAL** tail length, from anterior edge of cloaca to tip of tail

**TRL** trunk length, between axillary and groin regions

**VN** ventral scale count, number of ventral body scales in a straight line along medial axis between transverse gular fold and anterior edge of cloaca

Principal component analysis was used to test whether morphological differences exist between the potential new species and its closest relatives. Mensural data used in the analysis include SVL, TAL, HL, HW, SEL, FLL, HLL, TRL, and T4L. Pholidosis data used in the analysis include LS, IL, NSL, SOR, MD, and T4S. Data for *Diploderma laeviventre* and *D. yangi* were obtained from Wang et al. (2016, 2022). Principal component analysis was performed using the prcomp command in R 4.2.2 and plotted on a scatterplot using the ggplot2 package in R 4.2.2 (R Core Team 2022).

Total genomic DNA was extracted from liver tissues preserved in analytical pure ethanol. The mitochondrial gene NADH dehydrogenase subunit 2 (ND2) was amplified and sequenced by the Tsingke Biotechnology Co., Ltd using published primers (Wang et al. 2019a). Sequence assembly was performed using SeqMan in Lasergene 7.1 (Burland 2000). Fifty-three sequences from 48 other species of *Diploderma* and two sequences from two outgroups were obtained from GenBank (Table 1). Sequences were aligned using MAFFT 7.471 (Katoh and Standley 2013). The best-fit substitution models were chosen using the Akaike information criterion in ModelFinder (Kalyaanamoorthy et al. 2017). The technical computation methods for genetic distance calculation and Bayesian inference and maximum-likelihood phylogenetic analyses were the same as those in Liu et al. (2024), except that the model used for both Bayesian inference and maximum-likelihood analysis was GTR+F+I+G4, and the Markov chains were run for 5,000,000 generations.

**Table 1.** Specimen voucher numbers, localities, and GenBank accession numbers of the sequences used in this study.

**Species**	**Voucher**	**Localit**y	**Accession**
*Diploderma crypticum* sp. nov.	KIZ 20201009	Chayu, Xizang, China	PZ753912
*Diploderma crypticum* sp. nov.	KIZ 20201010	Chayu, Xizang, China	PZ753913
*Diploderma crypticum* sp. nov.	KIZ 20201011	Chayu, Xizang, China	PZ753914
*Diploderma crypticum* sp. nov.	KIZ 20201012	Chayu, Xizang, China	PZ753915
*Diploderma crypticum* sp. nov.	KIZ 20201013	Chayu, Xizang, China	PZ753916
*Diploderma crypticum* sp. nov.	KIZ 20201014	Chayu, Xizang, China	PZ753917
*Diploderma crypticum* sp. nov.	KIZ 20201015	Chayu, Xizang, China	PZ753918
*Diploderma angustelinea*	KIZ 029704	Muli, Sichuan, China	MT577930
*Diploderma aorun*	KIZ 032733	Benzilan, Yunnan, China	MT577938
*Diploderma batangense*	KIZ 09404	Zhubalong, Xizang, China	MK001412
*Diploderma bifluviale*	CIB-CB23JC04	Barkam, Sichuan, China	PV833285
*Diploderma bowoense*	KIZ 044757	Muli, Sichuan, China	MW506020
*Diploderma brevicauda*	KIZ 044305	Lijiang, Yunnan, China	MW506021
*Diploderma brevipes*	NMNS 19607	Taiwan, China	MK001429
*Diploderma chapaense*	ZMMU NAP-01911	Sa Pa, Vietnam	MG214262
*Diploderma daduense*	CIB-CB2021227	Hanyuan, Sichuan, China	PP539949
*Diploderma danbaense*	KIZ2022048	Danba, Sichuan, China	OQ378180
*Diploderma daochengense*	DC001	Daocheng, Sichuan, China	OP595621
*Diploderma donglangense*	KIZ2022057	Muli, Sichuan, China	OQ378185
*Diploderma drukdaypo*	KIZ 027627	Jinduo, Xizang, China	MT577950
*Diploderma dymondi*	KIZ 040639	Dongchuan, Yunnan, China	MK001422
*Diploderma fasciatum*	KIZ 040192	Daweishan, Yunnan, China	OM055800
*Diploderma flaviceps*	KIZ 01851	Luding, Sichuan, China	MK001416
*Diploderma flavilabre*	KIZ 032692	Baiyu, Sichuan, China	MT577916
*Diploderma formosgulae*	KIZ 044420	Deqin, Yunnan, China	MW506024
*Diploderma hutiao*	KIZ 028291	Hutiaoxia, Yunnan, China	MT577921
*Diploderma iadinum*	KIZ 027697	Yunling, Yunnan, China	MT577956
*Diploderma jiulongense*	KIZ2022086	Jiulong, Sichuan, China	OQ378190
*Diploderma kangdingense*	20210916	Kangding, Sichuan, China	OP595625
*Diploderma laeviventre*	KIZ 014037	Basu, Xizang, China	MK001407
*Diploderma laeviventre*	KIZ 027691	Basu, Xizang, China	MT577892
*Diploderma laeviventre*	KIZ 027692	Basu, Xizang, China	MT577893
*Diploderma limingense*	KIZ2022015	Yulong, Yunnan, China	OP428783
*Diploderma luei*	NMNS 19604	Taiwan, China	MK001433
*Diploderma makii*	NMNS 19609	Taiwan, China	MK001431
*Diploderma menghaiense*	KIZ2019068	Menghai, Yunnan, China	PQ629456
*Diploderma micangshanense*	KIZ 032801	Shiyan, Hubei, China	MK578665
*Diploderma nangunhe*	KIZ2024100	Gengma, Yunnan, China	PQ629458
*Diploderma panchi*	KIZ 032715	Yajiang, Sichuan, China	MT577946
*Diploderma panlong*	KIZ 040137	Miansha, Sichuan, China	MT577906
*Diploderma polygonatum*	NMNS 19598	Taiwan, China	MK001427
*Diploderma qiaojiaense*	KIZ2021101	Qiaojia, Yunnan, China	PP889934
*Diploderma qilin*	KIZ 028332	Deqin, Yunnan, China	MT577941
*Diploderma shuoquense*	KIZ2022004	Xiangcheng, Sichuan, China	OP428773
*Diploderma slowinskii*	CAS 214906	Gongshan, Yunnan, China	MK001405
*Diploderma slowinskii*	KIZ 027543	Fugong, Yunnan, China	MT577910
*Diploderma splendidum*	KIZ 015973	Yichang, Hubei, China	MK001418
*Diploderma swild*	KIZ 034914	Panzhihua, Sichuan, China	MN266299
*Diploderma swinhonis*	NMNS 19592	Taiwan, China	MK001419
*Diploderma tachengense*	KIZ2022028	Weixi, Yunnan, China	OQ378195
*Diploderma varcoe*	WK-JK 011	Yuxi, Yunnan, China	MT577903
*Diploderma vela*	KIZ 019299	Quzika, Xizang, China	MK001414
*Diploderma xinlongense*	20210907	Xinlong, Sichuan, China	OP595613
*Diploderma yangi*	SWFU 005410	Chayu, Xizang, China	OL449603
*Diploderma yangi*	SWFU 005411	Chayu, Xizang, China	OL449606
*Diploderma yangi*	SWFU 005412	Chayu, Xizang, China	OL449604
*Diploderma yangi*	SWFU 005414	Chayu, Xizang, China	OL449605
*Diploderma yulongense*	KIZ2022009	Yongsheng, Yunnan, China	OP428777
*Diploderma yunnanense*	CAS 242271	Longling, Yunnan, China	MK001408
*Diploderma zhaoermii*	KIZ 019564	Wenchuan, Sichuan, China	MK001425
*Laodracon carsticola*	NUOL.R.2022.01	Khammuone, Laos	OR544068
*Paracalotes kakhiensis*	KIZ 015975	Gongshan, Yunnan, China	MK001435

## Results

**Figure 1.**
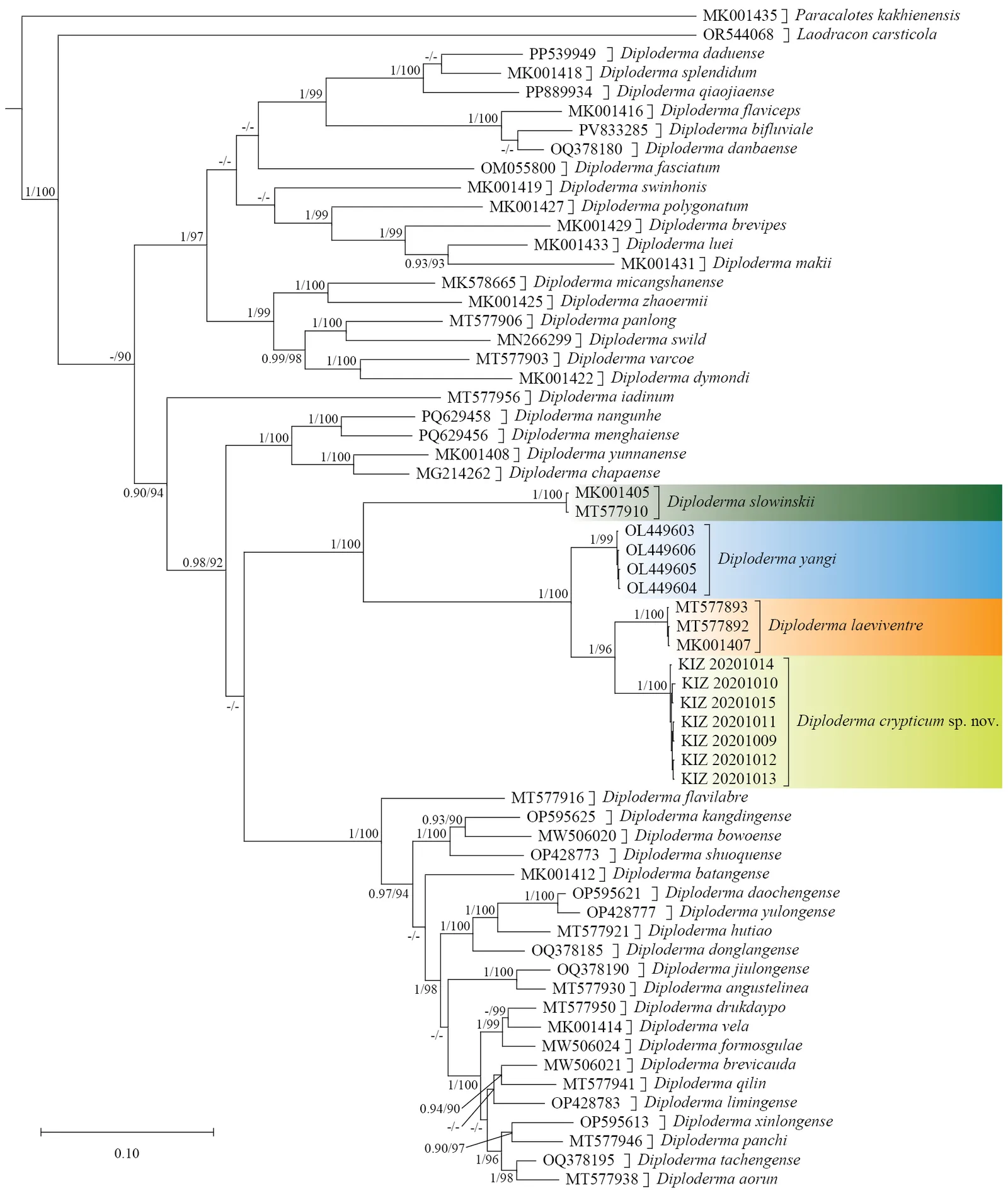
Bayesian inference phylogram of the genus *Diploderma* based on the ND2 gene. The four species inhabiting the Nujiang River system are highlighted in color. Numbers before slashes indicate Bayesian posterior probabilities, and numbers after slashes indicate the ultrafast bootstrap values of maximum-likelihood analysis. The symbol “–” represents values below 0.90/90.

The topologies inferred from Bayesian inference and maximum-likelihood analysis are consistent. All the specimens from the Nujiang River system were recovered as monophyletic, and the specimens from Wabu Village formed a distinct clade in this group sister to *Diploderma laeviventre* with strong support (Fig. 1). The average uncorrected pairwise distances between the sequences of the specimens from Wabu Village and *D. laeviventre*, *D. yangi*, and *D. slowinskii* were 4.8%, 6.5%, and 15.8%, respectively (Suppl. material 1).

**Figure 2.**
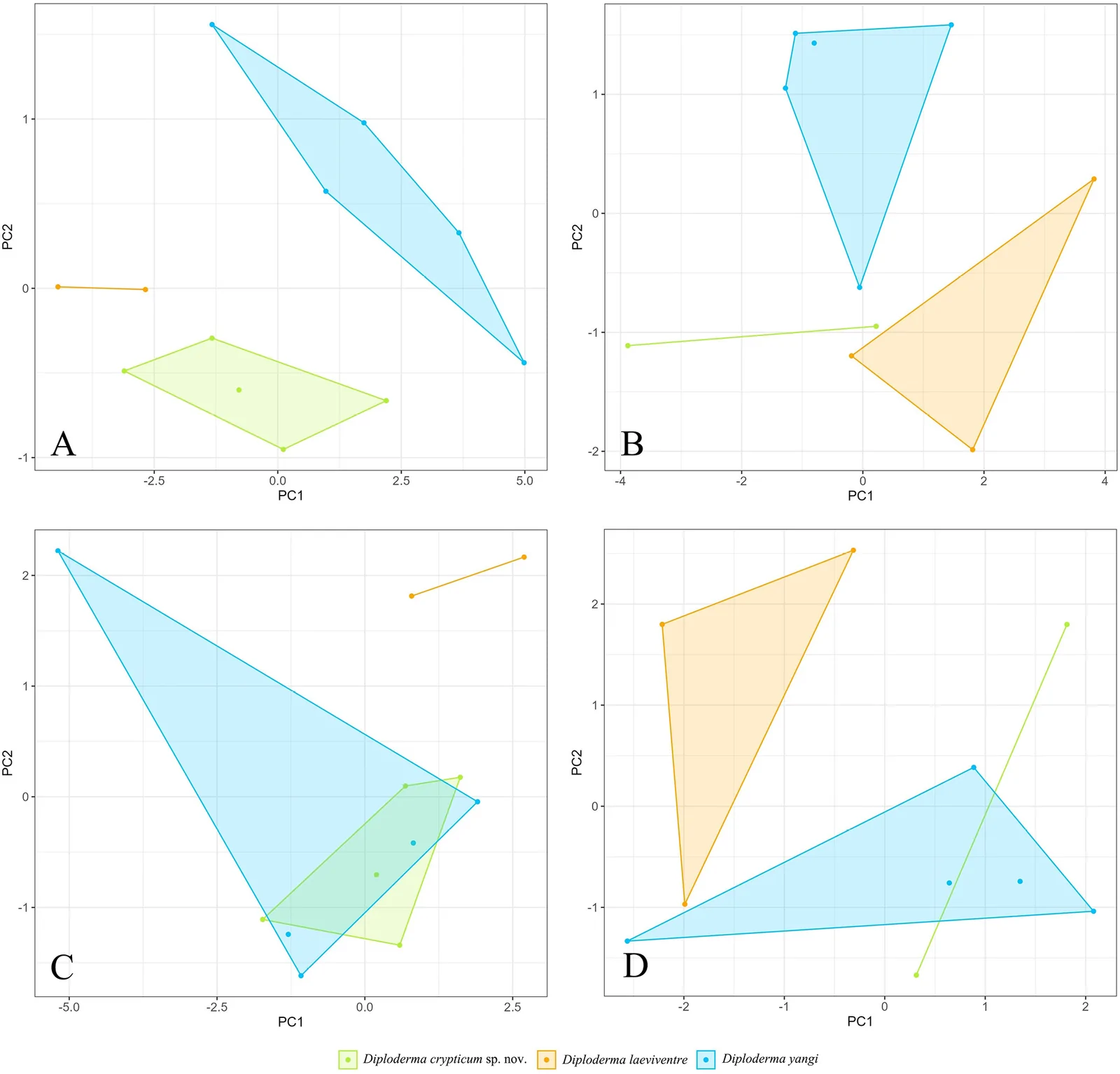
Scatterplot of principal component analysis. **A**. Mensural characteristics for males; **B**. Mensural characteristics for females; **C**. Pholidosis characteristics for males; **D**. Pholidosis characteristics for females.

The scatterplot based on the first two major principal components showed that the specimens from Wabu Village can be segregated from *Diploderma laeviventre* and *D. yangi* by mensural characteristics but overlap with *D. yangi* in pholidosis characteristics (Fig. 2).

### Taxonomy

#### Diploderma crypticum sp. nov.

Taxon classificationAnimaliaBoletalesDiplocystidiaceae

2057E405-7C95-5AC0-B48F-8E53D6795B16

https://zoobank.org/423F942F-7F79-4A75-B692-F17D0FCE398B

**Figure 3.**
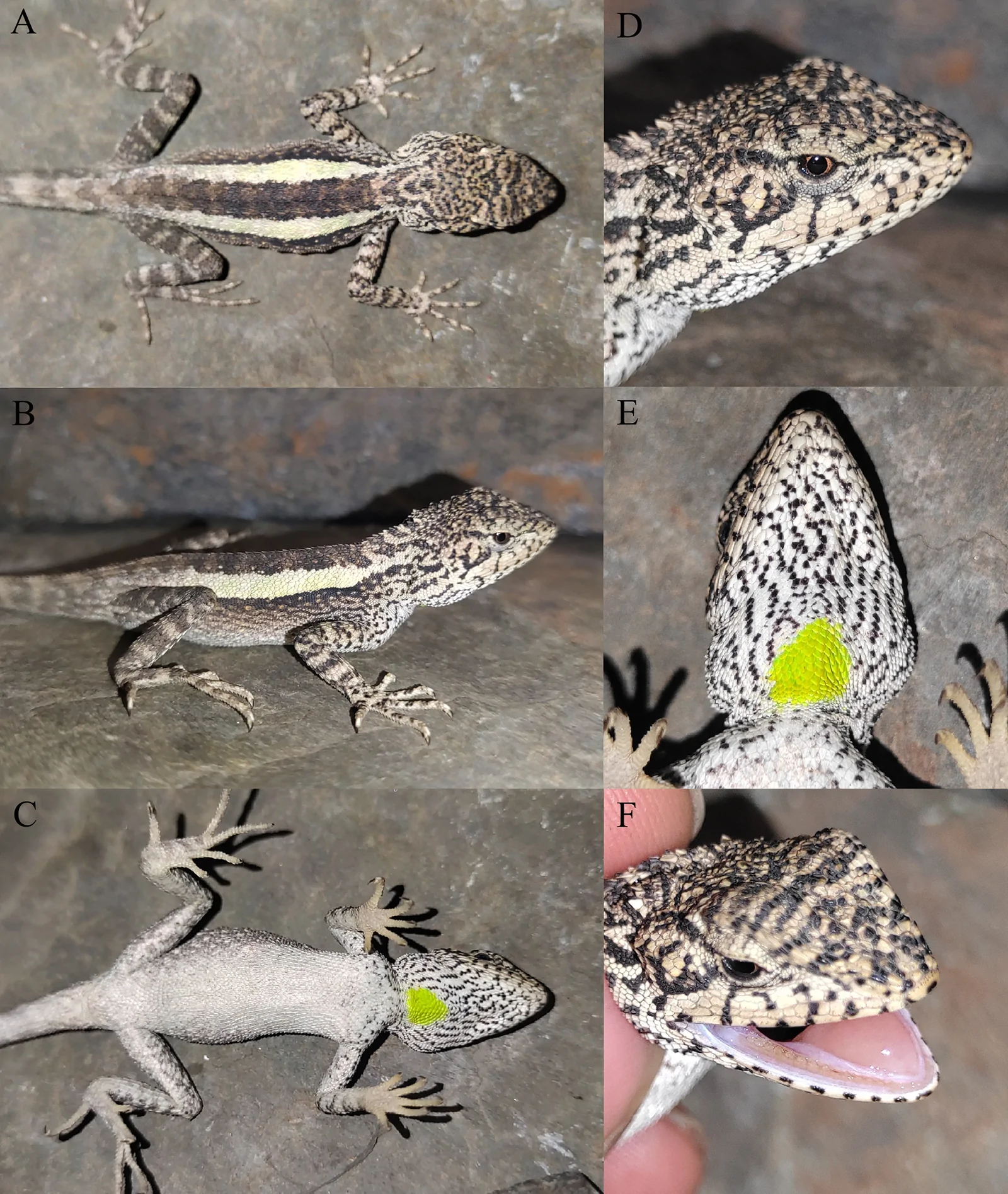
The holotype (KIZ 20201011) of *Diploderma crypticum* sp. nov. in life. **A**. Dorsal view; **B**. Lateral view; **C**. Ventral view; **D**. Close-up view of the lateral head; **E**. Close-up view of the ventral head; **F**. Close-up view of the oral cavity.

**Figure 4.**
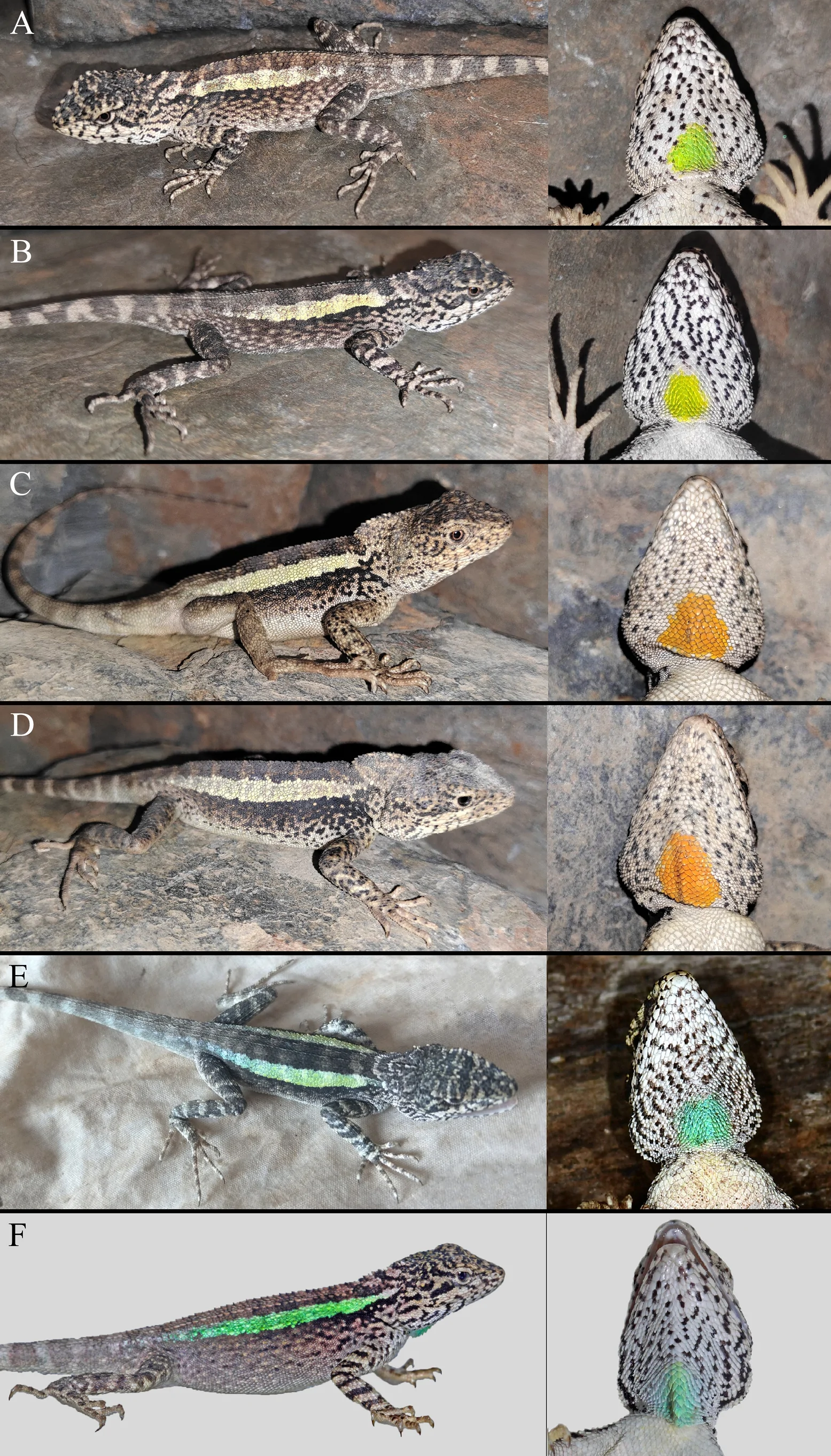
Comparison of the males. **A**. Paratype of *Diploderma crypticum* sp. nov., KIZ 20201012; **B**. Paratype of *Diploderma crypticum* sp. nov., KIZ 20201010; **C, D**. Topotypic specimens of *D. laeviventre*; **E, F**. Topotypic specimens of *D. yangi*.

**Figure 5.**
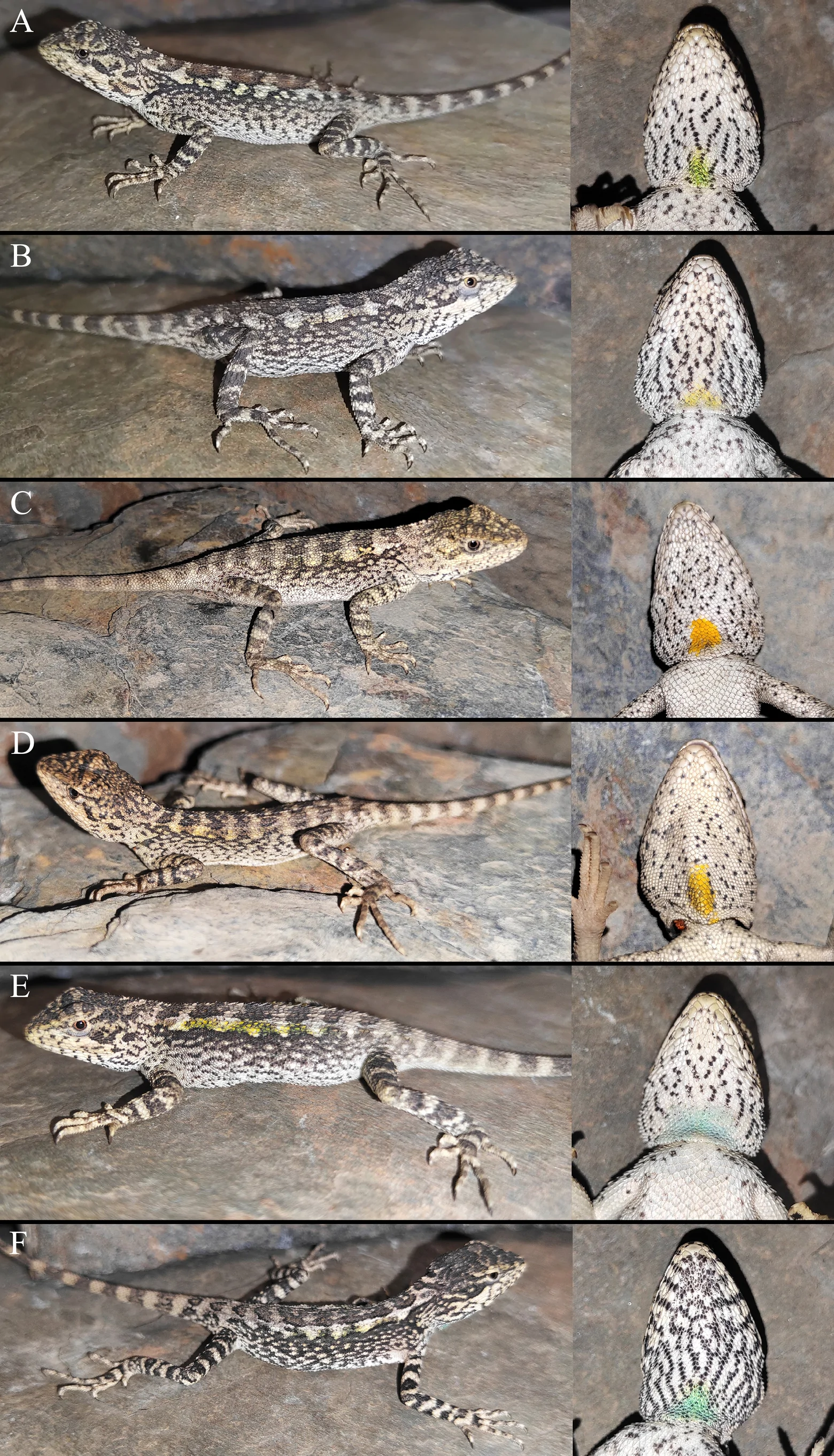
Comparison of the females. **A**. Paratype of *Diploderma crypticum* sp. nov., KIZ 20201015; **B**. Paratype of *Diploderma crypticum* sp. nov., KIZ 20201014; **C, D**. Topotypic specimens of *D. laeviventre*; **E, F**. Topotypic specimens of *D. yangi*.

Figs 3, 4A, B, 5A, B

##### Type material.

***Holotype***. KIZ 20201011, adult male, collected on 26 August 2020 by JiShan Wang from the valley of the Yuqu River, a tributary of the Nujiang River, in Wabu Village, Chawalong Township, Chayu County, Linzhi City, Xizang Autonomous Region, China (28°41'12"N, 98°26'14"E, 2500 m elevation). ***Paratypes***. KIZ 20201009–KIZ 20201010 and KIZ 20201012–KIZ 20201013, four adult males, and KIZ 20201014–KIZ 20201015, two adult females; collecting data the same as the holotype.

##### Etymology.

The specific epithet is a Latin adjective in the neuter gender, derived from the Greek *kryptos*, meaning hidden, named for the cryptic status of this species, and given in reference to the morphology similarity of the new species to *Diploderma laeviventre* and *D. yangi*.

##### Diagnosis.

*Diploderma crypticum* sp. nov. can be diagnosed from other species of the genus by a combination of the following characters: SVL 54.8–69.6 mm in males, 57.8–62.9 mm in females; TAL/SVL 1.95–2.10 in males, 1.91–1.95 in females; HL/SVL 0.30–0.32 in males, 0.30–0.31 in females; HW/HL 0.72–0.77 in males, 0.69–0.74 in females; SEL/HL 0.35 in males, 0.33 in females; FLL/SVL 0.44–0.47 in males, 0.46–0.47 in females; HLL/SVL 0.71–0.76 in males, 0.74 in females; T4L/SVL 0.17–0.18 in males, 0.18 in females; MD 50–56; VT 57–69; F4S 16–18, T4S 20–24; NSL mostly 1, rarely 2; tympanum concealed; nuchal and dorsal crest undeveloped; distinct transverse gular fold present; anterior ventral head scales smooth or feebly keeled, posterior ventral head scales strongly keeled; ventral body scales strongly keeled; dorsolateral stripes smooth edged and yellowish white in males; gular spot present in both sexes, yellowish green in males and yellowish green or light yellow in females; and oral cavity and inner lips light pink, tongue pink.

##### Description of holotype.

Adult male, body size moderate, SVL 65.0 mm; tail long, TAL 128.2 mm, TAL/SVL 1.97; limbs moderate, FLL 30.0 mm, FLL/SVL 0.46, HLL 47.7 mm, HLL/SVL 0.73. Head moderately long, HL/SVL 0.31, HW/HL 0.72; snout relatively short, SEL/HL 0.35. Rostral transversely elongated, bordered by ten scales, including two first supralabials; dorsal head scales heterogeneous in size and shape, all keeled; Y-shaped ridge present on dorsal snout. Nasal oval, separated from first supralabial by one row of scales on each side, separated from rostral by two small scales on each side; loreals small, keeled; three rows of scales between supralabials and orbit circle on each side, keeled; canthus rostralis elongated, greatly overlapping with each other; three enlarged, strongly keeled scales forming a ridge from posteroinferior eye to posterosuperior tympanum on each side; tympanum concealed under small scales; supralabials seven on each side, smooth. Mental approximately hexagonal, bordered by six scales, including two first infralabials; infralabials nine on each side, smooth; enlarged chin shields 11 on each side, smooth, first one connected to mental and first infralabial on each side, remaining ones separated from infralabials by one to three rows of scales on each side; ventral head scales heterogeneous in size, anterior and middle ones larger, posterior and side ones smaller, anterior ones smooth or feebly keeled, posterior ones strongly keeled; transverse gular fold present; gular pouch moderately developed.

Dorsal body scales heterogeneous in size and shape, all keeled, tip pointing backward; shoulder fold present; axillary scales much smaller than remaining dorsals; enlarged dorsal scales forming two intermittent longitudinal rows, one just above upper edge of dorsolateral stripe, another along lower edge of dorsolateral stripe on each side, remaining enlarged dorsal scales irregularly scattered on each side of body. Nuchal and dorsal crests undeveloped, continuous, no obvious erected skin fold under nuchal and dorsal crests; middorsal crest scales 51. Dorsal limb scales strongly keeled, almost homogeneous in size on forelimbs and heterogeneous in size on hind limbs; subdigital lamellae on fourth finger 16 on each side, subdigital lamellae on fourth toe 21 on left side and 20 on right side. Ventral body scales approximately parallel, almost homogeneous, all strongly keeled, ventral scales 66. Ventral limb scales all strongly keeled, smaller on upper arms and thighs and larger on forearms and crus. Tail scales all strongly keeled, ventral tail scales larger than dorsal tail scales.

##### Coloration of holotype in life.

Dorsal surface of head light brown with some indistinct black transverse stripes. Lateral surface of head light grayish brown, several indistinct radial black stripes around eye. Ventral surface of head white with many small black spots, gular spot yellowish green. Oral cavity and inner lips light pink, tongue pink.

Dorsal surface of body dark brown. Dorsolateral stripe yellowish white, edges smooth. Ventral surface of body grayish white.

Dorsal surfaces of limbs light grayish brown with several brownish black transverse stripes. Ventral surfaces of limbs grayish white with small grayish black spots.

Dorsal surface of tail light grayish brown with some transverse brownish-black bands. Ventral surface of tail grayish white.

##### Variations.

The paratypes are similar to the holotype in morphometric and pholidosis characteristics (Table 2). The coloration of the paratypes is also similar to that of the holotype except that the females have a relatively paler dorsal body color, grayish white discontinuous dorsolateral stripes, and a yellowish green or light yellow gular spot.

**Table 2.** Morphological and pholidosis data of the type specimens of *Diploderma crypticum* sp. nov. Measurements are in mm; see Materials and Methods section for abbreviations.

	**KIZ 20201011** ♂ **Holotype**	**KIZ 20201012** ♂ **Paratype**	**KIZ 20201013** ♂ **Paratype**	**KIZ 20201009** ♂ **Paratype**	**KIZ 20201010** ♂ **Paratype**	**KIZ 20201014** ♀ **Paratype**	**KIZ 20201015** ♀ **Paratype**
SVL	65.0	60.9	62.1	69.6	54.8	62.9	57.8
TAL	128.2	123.9	130.2	Incomplete	106.9	120.0	112.8
HW	14.4	14.5	14.3	16.3	12.4	14.2	11.9
HL	20.0	18.8	19.7	21.1	17.3	19.3	17.2
HD	10.0	10.0	10.3	11.9	9.0	10.6	8.9
SEL	6.9	6.5	6.8	7.3	6.1	6.3	5.7
FLL	30.0	26.9	29.2	31.1	25.4	28.9	27.0
HLL	47.7	43.2	47.0	49.9	41.7	46.8	42.8
T4L	11.4	10.6	10.8	11.7	10.1	11.1	10.5
TRL	27.3	27.5	26.6	31.7	23.1	28.0	25.4
SL	7/7	9/8	9/8	9/9	8/8	8/8	8/7
IL	9/9	10/10	10/9	9/10	9/10	10/9	8/8
CR	11/11	10/10	10/10	10/10	11/10	11/11	11/10
NSL	1/1	1/1	1/1	1/1	1/1	1/1	1/2
SOR	3/3	3/3	3/3	3/3	3/3	3/3	3/3
MD	51	53	51	53	56	50	54
VT	66	63	62	69	65	57	63
F4S	16/16	17/16	16/17	18/18	16/16	17/17	16/16
T4S	21/20	21/21	21/23	23/24	22/23	22/23	22/22
TAL/SVL	1.97	2.03	2.10	—	1.95	1.91	1.95
HL/SVL	0.31	0.31	0.32	0.30	0.32	0.31	0.30
HW/HL	0.72	0.77	0.73	0.77	0.72	0.74	0.69
SEL/HL	0.35	0.35	0.35	0.35	0.35	0.33	0.33
FLL/SVL	0.46	0.44	0.47	0.45	0.46	0.46	0.47
HLL/SVL	0.73	0.71	0.76	0.72	0.76	0.74	0.74
TRL/SVL	0.42	0.45	0.43	0.46	0.42	0.45	0.44
T4L/SVL	0.18	0.17	0.17	0.17	0.18	0.18	0.18

##### Comparisons.

*Diploderma crypticum* sp. nov. is phylogenetically closely related and most similar in morphological characteristics to *D. laeviventre* and *D. yangi*. However, *Diploderma crypticum* sp. nov. can be differentiated from *D. laeviventre* by having strongly keeled ventral body scales (vs smooth), a different coloration of the gular spot in life (yellowish green vs orange in males and yellowish green or light yellow vs orange or light orange in females) (Figs 4, 5), a relatively longer tail in females (TAL/SVL 1.91–1.95 vs 1.69–1.84), a relatively longer and wider head in females (HL/SVL 0.30–0.31 vs 0.28–0.29, HW/HL 0.69–0.74 vs 0.67–0.68), and fewer middorsal crest scales in both sexes (MD 50–56 vs 57–59); and can be differentiated from *D. yangi* by having a different coloration of the dorsolateral stripes in males in life (yellowish white vs green or bluish green), a different coloration of gular spot in life (yellowish green vs greenish blue in males and yellowish green or light yellow vs greenish blue or light blue in females) (Figs 4, 5), a relatively shorter tail in females (TAL/SVL 1.91–1.95 vs 1.97–2.09), a relatively shorter Toe IV in males (T4L/SVL 0.17–0.18 vs 0.20–0.21), and a relatively shorter snout in both sexes (SEL/HL 0.33–0.35 vs 0.38–0.45).

*Diploderma crypticum* sp. nov. differs from *D. brevipes* (Gressitt, 1936), *D. chapaense* (Bourret, 1937), *D. hamptoni* (Smith, 1935), *D. luei* (Ota, Chen & Shang, 1998), *D. makii* (Ota, 1989), *D. menghaiense* Liu, Hou, Wang, Ananjeva & Rao, 2020, *D. micangshanense* (Song, 1987), *D. nangunhe* Liu, Li, Yang, Hou, Rao & Ananjeva, 2024, *D. ngoclinense* (Ananjeva, Orlov & Nguyen, 2017), *D. polygonatum* Hallowell, 1861, *D. swinhonis* (Günther, 1864), and *D. yunnanense* (Anderson, 1878) by the presence of a transverse gular fold (vs absence); and differs from *D. dymondi* (Boulenger, 1906), *D. panlong* Wang, Che & Siler, 2020, *D. slowinskii*, *D. swild* Wang, Wu, Jiang, Chen, Miao, Siler & Che, 2019, and *D. varcoae* (Boulenger, 1918) by having concealed tympana (vs exposed).

*Diploderma crypticum* sp. nov. differs from *D. bifluviale* Liu, Wu, Zhang, Yang, Liu, Chen, Chang, Xie & Cai, 2025, *D. daduense* Cai, Liu, Liang, Hou, Zhou, Zhong, Li & Chang, 2024, *D. danbaense* Liu, Hou, Ananjeva & Rao, 2023, *D. drukdaypo* (Wang, Ren, Jiang, Zou, Wu, Che & Siler, 2019), *D. fasciatum* (Mertens, 1926), *D. flaviceps* (Barbour & Dunn, 1919), *D. qiaojiaense* Liu, Hou & Rao, 2024, *D. shuoquense* Liu, Hou, Rao & Ananjeva, 2022, and *D. vela* (Wang, Jiang & Che, 2015) by the presence of a colorful gular spot in life (vs absence).

*Diploderma crypticum* sp. nov. differs from *D. angustelinea* Wang, Ren, Wu, Che & Siler, 2020, *D. aorun* Wang, Jiang, Zheng, Xie, Che & Siler, 2020, *D. batangense* (Li, Deng, Wu & Wang, 2001), *D. bowoense* Wang, Gao, Wu, Siler & Che, 2021, *D. brevicauda* (Manthey, Denzer, Hou & Wang, 2012), *D. daochengense* Cai, Zhang, Li, Du, Xie, Hou, Zhou & Jiang, 2022, *D. donglangense* Liu, Hou, Ananjeva & Rao, 2023, *D. flavilabre* Wang, Che & Siler, 2020, *D. formosgulae* Wang, Gao, Wu, Dong, Shi, Qi, Siler & Che, 2021, *D. iadinum* (Wang, Jiang, Siler & Che, 2016), *D. jiulongense* Liu, Hou, Ananjeva & Rao, 2023, *D. limingensis* Liu, Hou, Rao & Ananjeva, 2022, *D. panchi* Wang, Zheng, Xie, Che & Siler, 2020, *D. qilin* Wang, Ren, Che & Siler, 2020, *D. tachengense* Liu, Hou, Ananjeva & Rao, 2023, *D. xinlongense* Cai, Zhang, Li, Du, Xie, Hou, Zhou & Jiang, 2022, and *D. yulongense* (Manthey, Denzer, Hou & Wang, 2012) by having a distinctly different coloration of the gular spot in males in life (yellowish green vs yellow, orange, blue, bluish green, sulfur, yellowish white, or pinkish red).

*Diploderma crypticum* sp. nov. differs from *D. grahami* (Stejneger, 1924) by having a much longer tail (TAL/SVL 1.91–2.10 vs. 1.64), having a distinct transverse gular fold (vs feeble), and the presence of dorsolateral stripes (vs absence); differs from *D. hutiao* Wang, Dong, Gao & Che, 2026 by having a different coloration of the ventrolateral body in males in life (white vs green) and the edges of the dorsolateral stripes being smooth in males (vs jagged); differs from *D. kangdingense* Cai, Zhang, Li, Du, Xie, Hou, Zhou & Jiang, 2022 by having a yellowish green gular spot in males in life (vs yellow or absent) and having a different coloration of the ventrolateral body in males in life (white vs yellow); differs from *D. splendidum* by having a different coloration of the dorsolateral stripes in males in life (yellowish white vs green or yellowish green) and having a yellowish green gular spot in males in life (vs yellow or absent); and differs from *D. zhaoermii* (Gao & Hou, 2002) by the edges of the dorsolateral stripes being smooth in males (vs jagged) and the absence of strongly erected skin fold under dorsal crest in males in life (vs presence).

##### Distribution.

**Figure 6.**
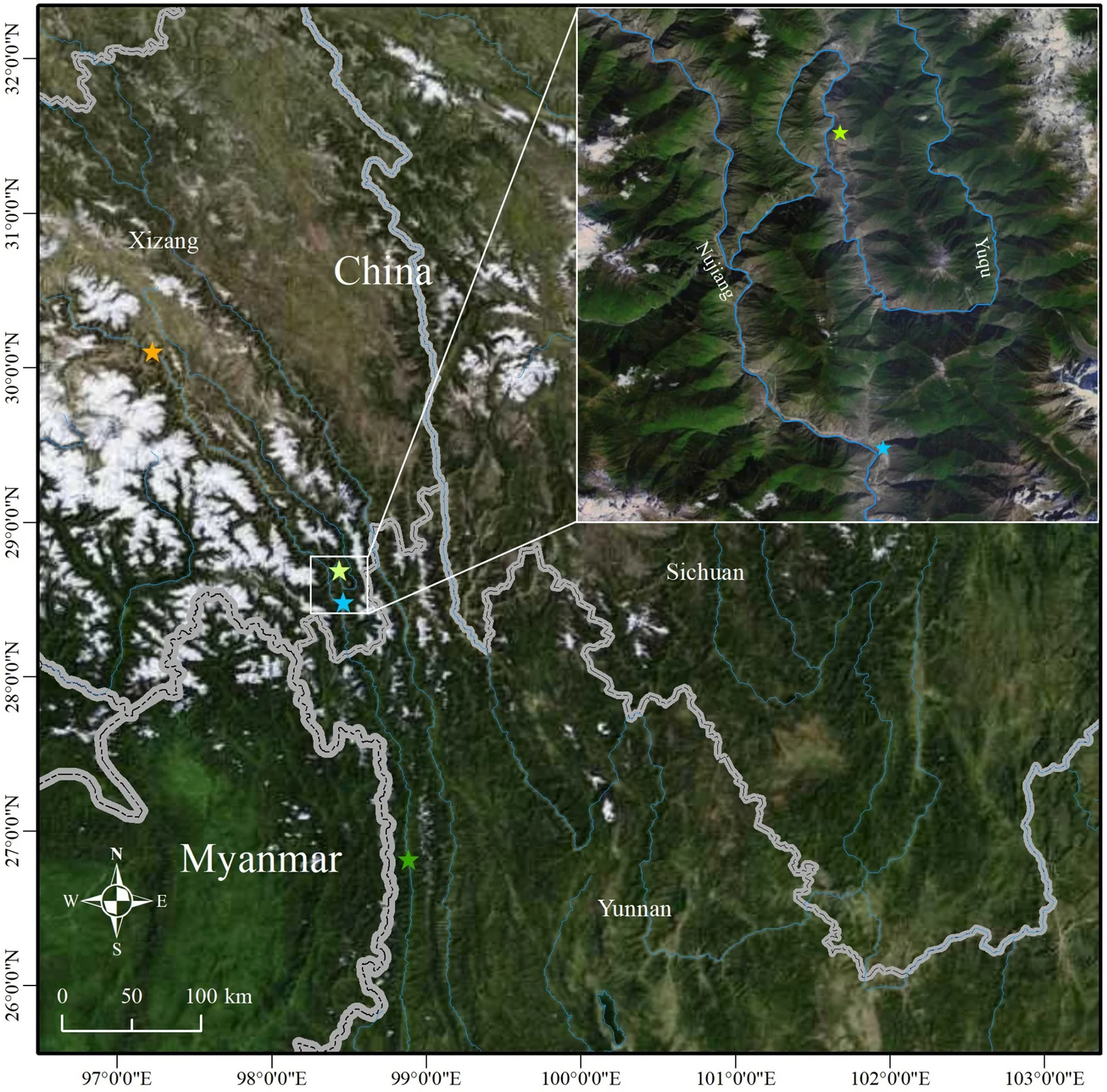
Map showing the type localities of the four *Diploderma* species inhabiting the Nujiang River system. Light green star: *Diploderma crypticum* sp. nov.; orange star: *D. laeviventre*; blue star: *D. yangi*; and dark green star: *D. slowinskii*.

This species is known only from the vicinity of Wabu Village, in Chawalong Township, Chayu County, Linzhi City, Xizang Autonomous Region, China (Fig. 6).

##### Natural history.

**Figure 7.**
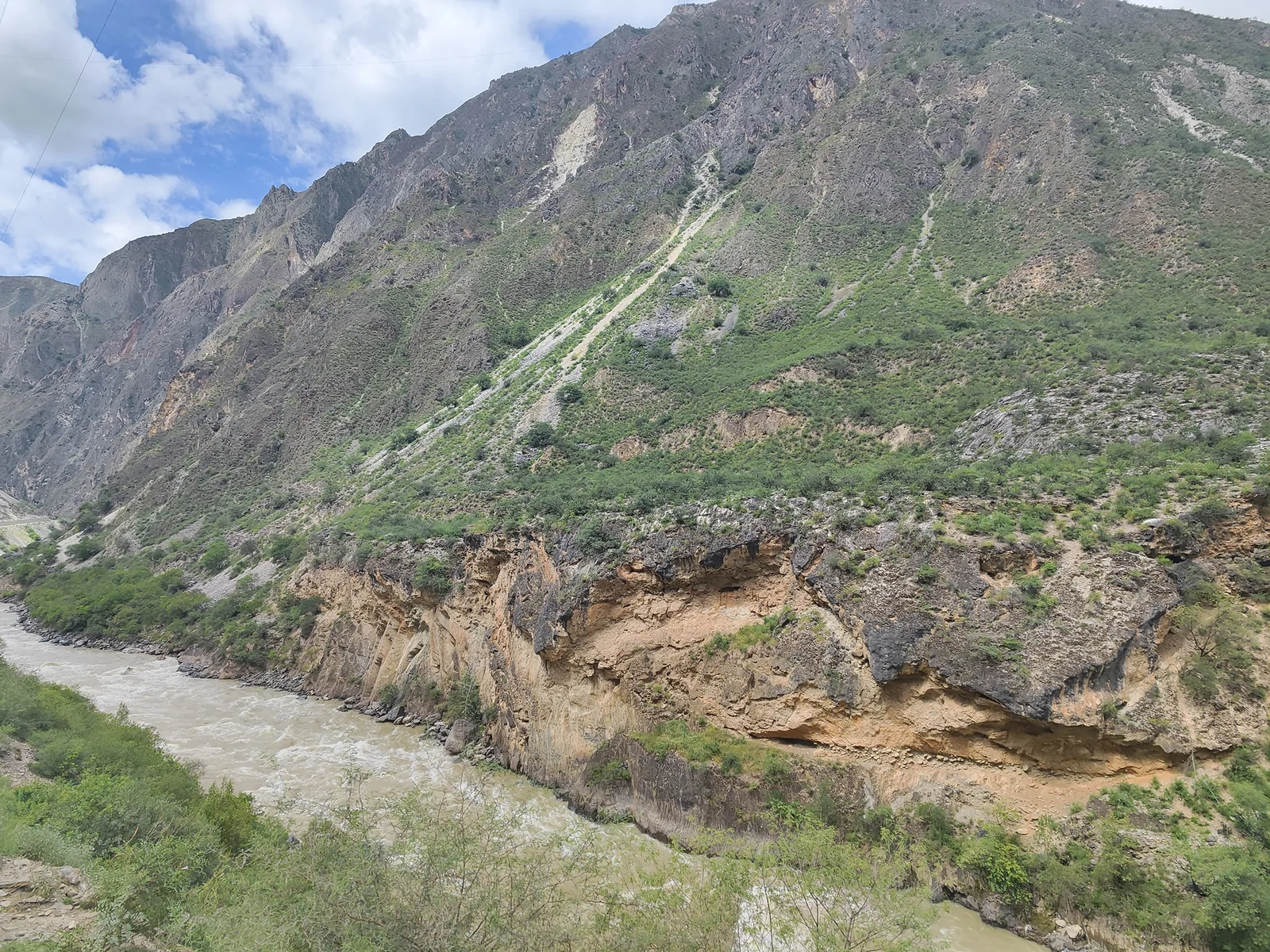
Habitat of *Diploderma crypticum* sp. nov. at the type locality.

This species is terrestrial and inhabits the hot, dry valley. The type locality has low shrubs and weathered rocks (Fig. 7). A road and a village are nearby. All specimens were collected during the day while they were basking on rocks. No gravid females were observed, and some juveniles were found during the survey, so it is inferred that their breeding season had already passed by August.

## Discussion

Species of the genus *Diploderma* can strongly change their body coloration. When they are stimulated or transferred to a non-normal living environment, their body coloration changes significantly. In a normal living environment and in a natural, relaxed state, they show the coloration we use for the description. Therefore, when we compare the body coloration of different species, we compare what they show most of the time in their normal living environment. For example, the dorsolateral stripes of males of *D. laeviventre* are yellowish white most of the time in their normal living environment, and when they are stimulated or transferred to an abnormal environment, the dorsolateral stripes will appear bluish green. However, we cannot say that the colors of the dorsolateral stripes of *D. laeviventre* and *D. yangi* are the same in males. Similarly, the color of the dorsolateral stripes of *Diploderma crypticum* sp. nov. is different from that of *D. yangi* in males. Although the dorsolateral stripes of males of *Diploderma crypticum* sp. nov. also appear bluish green when they are stimulated or transferred to an abnormal environment, the dorsolateral stripes of males of *Diploderma crypticum* sp. nov. are yellowish white most of the time in their normal living environment, while the dorsolateral stripes of males of *D. yangi* are bluish green most of the time in their normal living environment.

Although the body coloration of species of the genus *Diploderma* can change, the color of the gular spot is usually stable. The color of the gular spot usually does not change with stimulation or environmental changes. The original description of *D. yangi* includes four photos of this species in life. The gular spots of the individuals in the first two photos are light green, the gular spot of the individual in the third photo is greenish blue, and the gular spot of the individual in the fourth photo is not visible. We have conducted multiple field surveys at the type locality of *D. yangi*, and we found that all individuals have a greenish blue or light blue gular spot, but none has a light green gular spot. The first two photos showing light green gular spots in the original description of *D. yangi* were not taken in a natural background; they appear to have been taken indoors and have an overall yellowish tone. In contrast, the latter two photos were clearly taken outdoors in a natural environment. We suspect that lighting at the time of shooting distorted of the color of the first two photos, while the latter two photos present the true color under natural light. Therefore, the color of the gular spot of *D. yangi* in life should be greenish blue or light blue rather than light green.

The geographical distance between the type localities of *Diploderma crypticum* sp. nov. and *D. yangi* is only approximately 20 km; however, they are not in the valley of the same river (Fig. 6). The type locality of *D. yangi* is on the bank of the main stream of the Nujiang River, while the type locality of *Diploderma crypticum* sp. nov. is on the bank of the Yuqu River, which is a curved tributary of the Nujiang River. Although the two localities are close, high mountains block the direct line between them. Currently, *D. yangi* is known only from the valley of the main stream of the Nujiang River, while *Diploderma crypticum* sp. nov. is known only from the valley of the Yuqu River. These two species were not found to be sympatric.

Recently, Che and Wang (2026) treated *Diploderma yongshengense* as a synonym of *D. yulongense* and described the population previously considered *D. yulongense* as a new species, namely *D. hutiao*; they also regarded *D. jiulongense* as a subspecies of *D. angustelinea* and *D. kangdingense* as a synonym of *D. bowoense*. We agree that *D. yongshengense* is a synonym of *D. yulongense*, and we recognize the validity of *D. hutiao*. However, denying the validity *D. jiulongense* and *D. kangdingense* is too arbitrary, and we do not currently adopt this viewpoint. In addition, there is no obvious difference in morphology between *D. daochengense* and *D. yulongense*, and their genetic distance is very small, so we consider them conspecific. However, until the taxonomic status of *D. daochengense* is formally determined, we retain its validity for the time being.

The genus *Diploderma* previously contained 50 species, with 49 species recorded in China (Uetz et al. 2026). This study brings the total number of species in this genus to 51 and the number of species in China to 50. In addition, among the numerous species of this genus, only three species were previously recorded from the Nujiang River system (Wang et al. 2022; Uetz et al. 2026). The discovery of the new species increases the number of species inhabiting the Nujiang River system to four.

The population of *Diploderma crypticum* sp. nov. is relatively abundant at the type locality and nearby, but its habitat has been disturbed by human activities, such as landslides caused by road construction. We suggest strengthening the investigation of the population, distribution, and habitat of this species to assess its conservation status.

## Supplementary Material

XML Treatment for Diploderma crypticum

## Additional information

### Artificial Intelligence (AI) use

The authors accept full responsibility for the content of the manuscript, including the disclosure of any use of AI.

No AI tools were used in the preparation of this manuscript.

### Author ORCIDs

S. Liu https://orcid.org/0000-0001-7825-3006

J. Wang https://orcid.org/0000-0003-4660-163X

M. Hou https://orcid.org/0000-0002-3322-6570

D. Rao https://orcid.org/0000-0003-2680-1503

### Data availability

All of the data that support the findings of this study are available in the main text or Supplementary Information.

## Supplementary materials

Supplementary material 1 Uncorrected genetic pairwise distances between species based on the mitochondrial ND2 gene sequences Data type xls https://binary.pensoft.net/file/1829721This dataset is made available under the Open Database License (http://opendatacommons.org/licenses/odbl/1.0/). The Open Database License (ODbL) is a license agreement intended to allow users to freely share, modify, and use this Dataset while maintaining this same freedom for others, provided that the original source and author(s) are credited. Shuo Liu, JiShan Wang, Mian Hou, Wei Zeng, Hongyuan Zhang, Dingqi Rao
